# Development of potent HLA-A02:01-restricted peptide-based cytotoxic T-cells against SARS-CoV-2 infections in patients awaiting a kidney transplant

**DOI:** 10.3389/fimmu.2025.1664371

**Published:** 2025-10-06

**Authors:** Chih-Chao Chang, Ya Nan Liu, Zheng Xu, Elena-Rodica Vasilescu, Ping Li, Eric K. Ho, Muyang Li, Syed A. Husain, Govind Bhagat, Sumit Mohan, George Vlad, Nicole Suciu-Foca

**Affiliations:** ^1^ Department of Pathology and Cell Biology, Division of Immunogenetics and Cellular Immunology, Columbia University Irving Medical Center, New York, NY, United States; ^2^ Department of Medicine, Division of Nephrology, Columbia University Irving Medical Center, New York, NY, United States; ^3^ Department of Epidemiology, The Columbia University Renal Epidemiology Group, Columbia University, New York, NY, United States; ^4^ Department of Pathology and Cell Biology, Division of Hematopathology, Columbia University Irving Medical Center, New York, NY, United States

**Keywords:** SARS-CoV-2, COVID-19, T-cell therapy, tetramer, organ transplant, cell-mediated immunity, antigen specific-cytotoxic T cells, immunophenotyping

## Abstract

**Background:**

Controlling viral infections prior to solid organ transplantation is vital for successful engraftment and overall well-being of patients. One promising approach involves the deployment of viral antigen-specific cytotoxic T cells to eradicate viral pathogens. Although there have been attempts to develop anti-viral vaccines in the literature, the limited number of virus-specific cells which can be generated *in vitro* in the autologous system make it impracticable for autologous therapy.

**Methods:**

We developed a straightforward and scalable method for the *in vitro* expansion of SARS-CoV-2 Spike S1 peptide-specific cytotoxic CD8^+^ T cells. This was achieved through combinatorial stimulation with S peptide-conjugated polystyrene microspheres in the presence of cytokines IL-2, IL-7, and IL-15 for 14 days.

**Results:**

Using A2/S_269_-specific tetramers as markers, we compared induction of S-specific CD8^+^ T cells from patients awaiting kidney transplantation (n=67) with that of normal controls (n=65). We found that this method has the potential to achieve at least a 10-fold up to 200-fold increase in S-specific CD8^+^ T cells in 34.3% of kidney transplant candidates and 36.9% of normal controls, respectively. These SARS-CoV-2 specific CD8^+^ T cells released inflammatory cytokines, expressed effector-memory T cells markers, and killed target cells in a dose-dependent and antigen-specific manner.

**Conclusion:**

Our study demonstrates that viral antigen-specific cytotoxic CD8^+^ T cells can be robustly enriched *in vitro* from peripheral blood mononuclear cells of both healthy individuals and patients with kidney diseases using peptide-conjugated microspheres. Our findings provide novel insights into a potential therapeutic approach, using autologous anti-viral CD8^+^ T cells for transplant recipients/candidates.

## 1 Introduction

The COVID-19 pandemic, caused by the novel coronavirus SARS-CoV-2, has resulted in significant global mortality and disruption since early 2020, particularly affecting older adults and population with underlying health conditions. The swift development and rollout of mRNA vaccines targeting the virus’s Spike (S) protein have been effective in reducing its spread by generating robust humoral and cellular immune responses. However, the emergence of mutations in the S protein has led to a marked increase in breakthrough infections, which reached 19.1% among fully vaccinated individuals in New York State as of May 2023 (1). This situation poses even greater risks for immunocompromised individuals, including patients who required solid organ transplants (SOT) and allogeneic stem cell transplants (ASCT), representing nearly half of the breakthrough hospitalizations (2, 3).

The clearance of the virus following SARS-CoV-2 infection, a vital step in recovery, has been shown to correlate more closely with the activation of S-specific CD8^+^ T cells than with the presence of S-specific neutralizing antibodies or N-specific CD8^+^ T cell activation (4, 5). These findings underscore the critical role of antigen specific CD8^+^ cytotoxic T cells, which persist longer than neutralizing antibodies in both infected and vaccinated individuals, in eradicating SARS-CoV-2 viruses. Recent studies have highlighted the persistence and protective capacity of virus-specific CD8^+^ T cells, even in the absence of robust antibody responses (6).

Despite advances in antiviral drug development, limitations such as drug resistance, toxicity, and incomplete viral clearance persist, motivating the exploration of alternative immunotherapeutic avenues. Among these, the adoptive transfer of antigen-specific cytotoxic T cells has emerged as a promising intervention to target persistent or refractory viral infections in immunocompromised individuals (7). This strategy hinges on the ability to generate or expand robust populations of virus-specific T cells capable of recognizing and eliminating infected cells with high precision.

Adoptive transfer of allogenic-antigen specific T cells against opportunistic viral infections, particularly by cytomegalovirus (CMV), BK virus (BKPyV), and JC virus (JCPyV) in immunocompromised individuals have been well studied (8–10). Both antiviral therapies (e.g., ganciclovir) and the adoptive transfer of the third-party virus-specific cytotoxic T lymphocytes (CTLs) have been utilized extensively, each offering distinct advantages and challenges (11).

However, generating enough functional, autologous virus-specific cytotoxic T cells from immunocompromised individuals has proven challenging. Factors like immunosuppression, underlying disease, and prior treatments may limit the frequency and functionality of these cells in transplant candidates. Thus, there is a pressing need for scalable, reproducible protocols to efficiently enrich and expand antigen-specific T cells suitable for therapeutic use, especially in the context of emerging pathogens such as SARS-CoV-2.

Attempts to develop antigen-specific cytotoxic T-cells for SARS-CoV-2 therapy have been performed with some success. Recent findings from two small Phase I clinical trials indicate that cytotoxic T cells, generated *in vitro* from healthy donors who have recovered from COVID-19, can be safely and effectively utilized to treat high-risk ambulatory COVID-19 patients (12, 13). In this study, we focused on the HLA-A*02:01-restricted antigen epitope S_269-277_, referred to as A2/S_269_, which is conserved across all SARS-CoV-2 variants and has been relatively well studied (14–16). Using A2/S_269_-specific tetramers as markers, we developed a straightforward and scalable method for the *in vitro* expansion of SARS-CoV-2 Spike S1 peptide-specific cytotoxic CD8^+^ T cells in both normal controls and kidney patients. This study lays the groundwork for a novel and personalized approach to adoptive T cell therapy using a patient’s virus-specific T cells (VSTs), offering a promising strategy for enhancing antiviral immunity in immuno-compromised populations. By leveraging advances in peptide-based stimulation and culture systems, it is now feasible to selectively expand populations of cytotoxic T cells targeting conserved viral epitopes, opening new avenues for personalized immunotherapy in at-risk patient populations.

## 2 Materials and methods

### 2.1 Reagents

FluoSpheres^®^ were from Thermo-Fisher Scientific. Flow cytometry reagents, including CD27-PE-Cy7, CD28-PerCP -Cy5.5, CD69-Alexa Fluor 488, CD95- BV421 and other Flow Cytometry reagents were purchased from BD Biosciences. TaqMan^®^ probes and Superscript^®^ III first -strand synthesis for RT-PCR system were ordered from Thermo-Fisher Scientific. Peptides (9-mers) were synthesized by GenScript^®^. Tetramer PE-SARS-CoV-2 S (YLQPRTFLL _269-277_), and tetramer PE-CMV pp65 (NLYPMVATV (495–503), and a photosensitive biotinylated monomer, HLA-A*02:01 KILGFVFJV were generously provided by NIH Tetramer Core Facility (Emory University, GA).

### 2.2 Human subjects, patients’ privacy protection and sample collections

Patients (n=67) awaiting kidney transplants or prospective kidney donors (n=51), who came to CUIMC for patient care from September 2022 to January 2025, were identified through our medical record system. Plasma and PBMCs prepared from these individuals were stored at -80°C and liquid nitrogen, respectively, prior to being used for the study. High resolution HLA typing was used to confirm if subjects were either heterozygous or homozygous for HLA-A*02:01. Immunological status of subjects with respective to COVID-19, besides the records of SARS-CoV-2 previous infection and/or COVID-19 vaccination, was confirmed by Multiplexed magnet bead-based anti-SARS-CoV-2 IgG assay as described in the next Section.

### 2.3 Multiplexed magnet bead-based assay for detection of IgG antibodies against SARS-CoV-2’s viral antigens

Bio-Plex Pro Human IgG SARS-CoV-2 Serology Assay System (Bio-Rad) for simultaneous detection of viral proteins (RBD, S1, S2 and nucleocapsid (N) proteins were previously described (17, 18). Sample data (Medium Fluorescence Intensity, MFI) were acquired using xPONENT^®^ Software and analyzed by Microsoft Excel Software. Sample MFI above the cutoff (for Nucleocapsid (N),>2,000, for RBD, Spike 1 and Spike 2, > 6,800) were counted as positive for respective IgG antibodies. Subjects positive for both anti-N and anti-S1 IgG antibodies were counted as previous SARS-CoV-2 infected with or without COVID-19 vaccination. Subjects seropositive for Spike I IgG but were seronegative (MFI <1 ,000) for anti-N were as counted as COVID-19 vaccinated but SARS-CoV-2 uninfected.

### 2.4 Preparation of HLA-A*02:01 peptide conjugated spheres and priming autologous PBMCS

Carboxylate-modified polystyrene yellow/green Fluorescent (1.0 μm in size) and Blue Fluorescent (1.0 μm in size) FluoSpheres were purchased from Thermo-Fisher Scientific. For peptide-bead conjugation, 300 μl HLA-A*02:01 restricted peptides (at 1μg/ml) were added to MES buffer for a final concentration of 50 mM MES PH=6.0. After extensive vortex, 150 μl spheres (about 6.3x10^9^ beads) were added to the peptide solution as described above and incubated for 15 min at room temperature. Three hundred μl freshly prepared conjugation reagent, EDAC (10 mg/ml Thermo-Fisher Scientific), were then added to the mixture of peptide-Spheres with extensive vortex, incubated at room temp for 4 hours, or overnight with constant rotating. The glycine solution was added at the end of incubation to a final concentration of 100 mM and incubated for another 30 min at room temperature with rotating to stop EDAC reaction. Conjugated beads were collected at 4°C at 14,000 RPM centrifugation for 30 min, washed with PBS 3 times and resuspended in original volume (750 μl) with 1% BSA-PBS in the presence of Sodium Azide (0.4 nM). The degree of conjugation was monitored by Flow Cytometry using BD FACS(Lyric™) Analyzer. We typically used 10ul ( ∼1x10^8^ beads) of peptide-conjugated beads or control (non-peptide conjugated) beads per 10^6^ cells for a 100:1 bead/cell ratio. Priming of PBMCs were described in Results Section.

### 2.5 Endocytosis inhibition assays

Frozen PBMC were thawed and washed with RPMI complete medium and were seeded on a 96-well flat-bottom plate at 1x10^5^/250 μl in complete medium for 30 minutes at 37°C prior to pre-treatment with cytochalasin D (Fisher Scientific) at various concentrations (1, 1.3, 10, 30 mM) for another 30 min at 37°C. Polystyrene Fluorescent (yellow-Green 505/515 nm), (1.0 mm in size) sphere beads at 100x excess were then added to cells and incubated for additional 1h at 37°C. Cell-beads mixtures were washed twice with a wash buffer (2 mM EDTA 0.5% BSA in PBS), measured by Flow Cytometric analyses.

### 2.6 Tetramer synthesis and staining, flow cytometric analyses of surface and intracellular markers

PE-SARS-CoV-2 (ORF1ab_(2332-2340),_ hereafter referred to as PE-A2/ORF1ab_2332_ was prepared by ligand exchange between HLA-photosensitive A*02:01 KILGFVFJV monomer and peptide ILFTRFFYV (19) followed by tetramerization with PE-streptavidin using the protocol provided by Toebes et al (20). Detection of tetramer^+^CD8^+^ T cells was performed by culturing with Dasatinib (Sigma-Aldrich) at a final concentration of 50 μM at 37°C for 30 min, followed by immediate incubation with tetramer PE-A2/S_269_ (at 1:1,000) or PE-A2/CMV pp65_495_ (at 1:2,000) or PE- A2/ORF1ab_2332_ (at 1:2,000) for 1 h at 4 °C in the dark without washing. After tetramer staining, cells were washed with FACS buffer, stained with surface markers including APC-CD8 (clone HIT8a) along with other surface markers for 20 min at 4 °C.

Staining of intracellular cytoplasmic proteins was performed by treatment of cells with 100 μl IC fixation buffer (Invitrogen) for 40 min at 4 °C, followed by two washes with permeabilization Buffer (Invitrogen) for 10 min prior to staining with Perforin-PerCP-CY5.5 (BD Biosciences) at 4 °C for 30 min. After washing with 0.5 ml FACS buffer, cells were acquired on a BD FACS Lyric™ Cytometer and analyzed by FCSExpress™ Software. Multitest™ 6-ColorTBNK (BD Biosciences) antibodies were used to determine major lymphocyte subsets after 2 weeks of *in vitro* stimulation with peptide-spheres and cytokines as described in manufacturer’s protocol.

### 2.7 ELISPOT and Luminex based ProcartaPlex assays for detection of IFN-γ, TNF-α

The IFN-γ ELISPOT pair (capture and detection antibodies) kit was purchased from BD Bioscience whereas the immunodetection system was purchased from ImmunoSPOT™ (Ohio, USA). ELISPOT plates (Sigma-Aldrich) were first activated, coated with capture antibodies (5μg/ml), and blocked with 10% FBS-RPMI medium for 2 h prior to the experiment. Serum free medium (100 μl) in the presence and absence of cognate peptides (40 μg/ml) or 0.2x concentration of Phytohemagglutinin (PHA) solution were first added to an ELISPOT plate. *In vitro* activated (see above) or naïve (not activated) cells at 5x10^4^, were washed extensively, and added to the corresponding wells. After 18 h incubation at 37 °C, plates were washed four times with 0.05% Tween20-PBS prior to addition of anti-human IFN-γ detection antibodies (2μg/ml) and incubated for 2 h at room temperature with shaking. The spots were detected and developed by streptavidin-AP and Blue Developer Solution™. Visualization and quantitation were performed by ImmunoSPOT 2™ software.

For detection of cytokine release from supernatants of *in vitro* activated cells re-stimulated with cognate peptide, Luminex based combinable ProcartaPlex™ (Invitrogen) assay systems were used. Magnetic beads conjugated with capture mAbs recognizing IFN-γ and TNF-α were pre-mixed and added to a clear flat bottom 96-well plate, washed 3 times on a handheld magnetic stand, followed by incubation with 50 μl supernatant obtained from cell cultures of *in vitro* stimulated cells for 2 hours at room temperature with shaking (600 rpm). After incubation, beads were washed 3 times prior to incubation with biotinylated detection antibody for another 30 min at room temperature with shaking, followed by 3 washes and incubation with Streptavidin-PE. Beads were then washed and resuspended in Reading Buffer prior to being acquired by FLEXMAP 3D™ and analyzed by Microsoft’s Excel Software.

### 2.8 Realtime PCR gene expression analysis

To analyze RNA profile of A/S_269_^+^ CD8^+^ T cells, PBMCs from normal controls were stimulated *in vitro* with S_269-277_-peptide conjugated Spheres in the presence of cytokines for 2 weeks as described. Tetramer^+^ CD8^+^ cells and tetramer^-^ CD8^+^ T cells were sorted by BD FACSAria™ III Neptune Cytometer and analyzed by FACSDiva 9.0.1 software. Fresh sorted cells were immediately lysed in RNeasy (Qiagen) RPE buffer supplemented with β-mercaptoethanol; total RNA from cells was then purified and stored at -80 °C. The first strand cDNA was synthesized from total RNA using Superscript III System (Invitrogen). Real-time PCR was performed using proprietary TaqMan gene expression probes (Applied Biosystems, Foster City, CA) and was run in duplicate in a Roche480 with a 384-well plate. Data was collected and analyzed for Realtime PCR measurement of gene expression. A housekeeping gene, GAPDH, was used as an internal reference.

### 2.9 Cell mediated cytotoxicity assay

We employed T2 (ATCC CRL-1922) as the target cells for HLA-A*02:01 restricted cytotoxicity assays. T2 cells were pulsed with either 20 μg/ml S_269–277_ peptide or 20 μg/m CMVpp65_495–503_ for 18 h at 37°C. The target cells were then washed twice and labeled with Calcein AM (Invitrogen) for 30 min whereas A2/S_269_^+^ T effectors generated *in vitro* (see above) were labeled with CellTrace Violet (Invitrogen) for 30 min at room temperature. Labelled target cells and effector cells were washed twice and adjusted to 5x10^5^/ml and 5x10^6^/ml in the medium containing IL2/IL7/IL15, respectively. Target cells at 2x10^4^ were added to wells of a 48-well plate followed by addition of effectors at different ratios (30:1, 15:1 7.5:1). Target cells alone and effectors alone were also placed in the plate as negative controls. The plate was briefly centrifuged for 1 min and incubated at 37 °C for 5 h. At the end of incubation, cells were washed twice and stained with Ethidium (Red) homodimer-1 (Invitrogen) at room temperature for 10 min, washed once prior to acquiring and analyzed by NovoExpress Software in NovoCyte Penteon (Agilent) Cytometer.

### 2.10 Statistical analyses

Comparative analyses and generation of the graphs were carried out using GraphPad Prism 10.04. Normally distributed data are presented as mean ± S.D., while data that were not normally distributed were presented as median ± interquartile range (Q1-Q3). Categorical data are reported as numbers and percentages. Mann-Whitney U test is used to compare two nonparametric groups. The Spearman correlation coefficient r was calculated for quantifying the association between continuous variables. Two-tailed *p* values were reported with *p <*0.05, considered significant.

## 3 Results

### 3.1 Cell stimulation protocol

**Figure 1:**
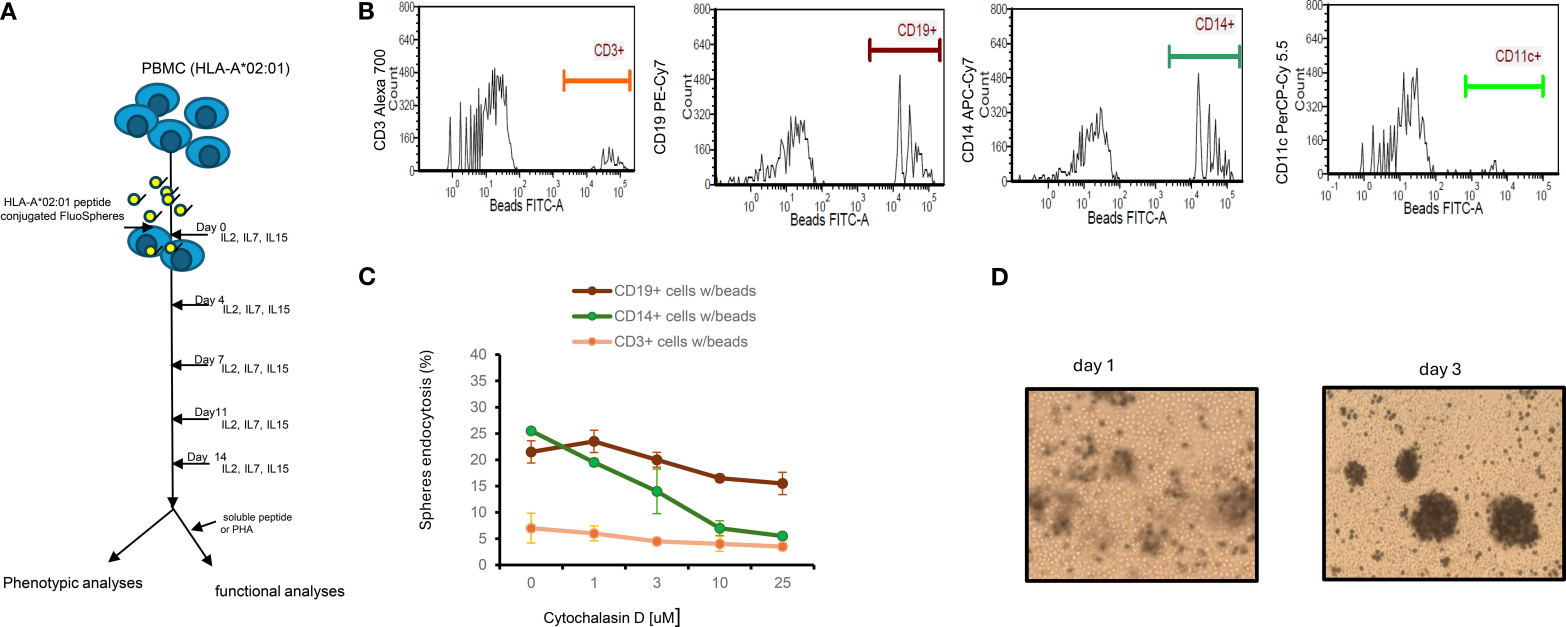
Experimental design, mechanism of endocytosis and cytokine dependent growth of lymphocyte. **(A)** Experimental design: Thawed PBMCs (1-3x10^6^) carrying HLA*02:01 genotype were cultured with peptide conjugated spheres (either yellow-green (365/415) nm or blue (505/515) at a ratio of 1:100 ratio in the presence of IL2/IL7/IL15 for 14 days. Half of the culture medium was replaced every 4 days. Cells were analyzed for the appearance of tetramer^+^ or surface markers; in some experiments, cell cultured supernatants were also collected for other assays such as IFN-γ release. **(B)** Endocytosis of FluoSpheres by cells from PBMCs. Thawed PBMCs (n=3) were cultured with yellow-green (detected in FITC channel) Spheres in the same culture medium as described for 1, 4 and 12 hours. Cells were then washed extensively and counter-stained with CD3, CD14, CD19 and CD11c mAbs prior to analyses by Flow Cytometry. A representative result of cells from one PBMCs after 1 h of co-culture was shown. **(C)** Inhibition of endocytosis of FluoSpheres by cytochalasin D in monocytes is dose dependent. The results are for one-hour treatment, which includes 30 min of pre-treatment and 30 min of treatment with cytochalasin D, are Mean ± S.D. of two experiments. **(D)** Snapshot (100X) of cells/beads co-cultures. PBMC cells at 1x10^6^ and beads at 1x10^8^ were co-cultured in a 48-well-plate containing 1ml medium supplemented with IL2/IL7/IL15. Photos were taken on day 1 and day 3. .

Preparation of antigen peptide-conjugated spheres was detailed in Materials and Methods. Peptide conjugated spheres, along with control (unconjugated) spheres were used to stimulate thawed peripheral blood mononuclear cells (PBMCs) from donors with homozygous or heterozygous HLA-A*02:01 genotypes. The cultures were maintained for two weeks in the presence of cytokines IL-2 IL-7 and IL-15 (**Figure 1A**). It has been previously reported that combinations of IL-2, IL-7, and IL-15 can induce robust proliferation of both antigen-independent (21) and antigen-specific (22) CD8^+^T cells. Cell-bead aggregates, which formed after one day of culture, significantly increased in size by day three (**Figure 1B**). This growth indicates that cells were recruited to create a three-dimensional structure composed of beads and cells. Such cell-bead aggregates, often observed in co-cultures with polystyrene or silica beads (23), have been suggested to promote cell proliferation and differentiation (24). At the end of the culture period, we analyzed the composition of the expanded cell populations using 6-color TBNK- flow cytometry assays (BD Biosciences). The predominant cell population was CD8^+^ T cells (51.0 ± 12.9%), followed by CD4^+^T cells (35.3 ± 17.4%) and CD16^+^CD56^+^ natural killer cells (3.7 ± 1.7%), across six independent lines (data not shown). Notably, depletion of NK cells from PBMC resulted in only a modest reduction of NK cells in the final cultures (2.7%).

### 3.2 Uptake of peptide-conjugated FluoSpheres by B lymphocytes and monocytes in cryopreserved PBMCs via phagocytosis

Efficient priming of peripheral blood-derived T cells requires antigen presentation by either professional or non-professional antigen-presenting cells (APCs). To identify which cell types of peripheral blood mononuclear cells (PBMCs) internalize peptide-conjugated spheres, we incubated yellow-green FluoSpheres (detected in the FITC channel) with cryopreserved PBMCs at a 100:1 bead-to-cell ratio for varying durations (1, 4, and 12 hours). Following 1 hour incubation, cells were extensively washed and stained with monoclonal antibodies against CD19 (B lymphocytes), CD14 (monocytes), CD3 (T lymphocytes), and CD11c (dendritic cells/professional APCs). Samples were then analyzed via flow cytometry. As shown in **Figure 1C**, after 1 hour of incubation, FluoSpheres were internalized by: 21.5± 2.1% of CD19^+^ B lymphocytes, 25.5 ± 0.7% of CD14^+^ monocytes, 5.0 ± 2.8% of CD3^+^ T lymphocytes, and 0.2 ± 0.2% of CD11c^+^ dendritic cells. Bead retention remained stable up to 12 hours (data not shown).

Since CD11c^+^ dendritic cells are known to be extremely sensitive to cryopreservation, only an exceptionally low percentage of these cells may contain beads. The high uptake by B lymphocytes and monocytes indicates their significant role in internalizing peptide-conjugated spheres under these conditions. An insignificant number of bead-uptakes by CD3^+^ T cells may reflect non-specific bead binding due to their abundance in PBMCs.

To explore the mechanism of bead internalization, we evaluated various endocytosis inhibitors. When Cytochalasin D (3 µM), an inhibitor of F-actin polymerization (25), was added to cultures for 1 hour, bead uptake by monocytes was reduced by over 50% (**Figure 1D**) in a dose-dependent manner. This suggests that internalization of spheres is actin polymerization-dependent, implying that phagocytosis is the primary mechanism for microsphere uptake by monocytes. This aligns with the general understanding that particles ≥1 µm are mainly internalized via phagocytosis, a process significantly more efficient than passive peptide diffusion. B lymphocytes, despite having the same capacity to internalize FluoSpheres as monocytes in our study, were less sensitive to Cytochalasin D at the same concentration, implying that they may use a distinct uptake mechanism, possibly through B cell receptors (26).

We also tested Dynole 34-2™ (Cayman Chemical), a well-characterized Dynamin inhibitor (27), at 10 µM to assess the role of Dynamin-dependent endocytosis. We found no reduction in uptake by either B lymphocytes or monocytes (data not shown), suggesting that clathrin-mediated endocytosis (CME) and fast endophilin-mediated endocytosis (FEME), both of which require Dynamin GTPase activity (28), are unlikely to be involved in this context.

### 3.3 Cohorts and patient characteristics

For this study, we recruited a total of 133 subjects. Eligible participants were categorized into two cohorts: the kidney patient group (transplant candidates) (n=67) and the normal control group (n=66), consisting of 51 prospective kidney donors and 15 additional blood donors. Kidney patients in our cohort were significantly older (median age 64.0, IQR: 52-68) and predominantly male, while prospective kidney donors were younger (median age 49.0, IQR: 37-60) and predominantly female. Most patients (>95%) were either in advanced stages (III to V) of chronic kidney disease (CKD) or already with End-Stage Renal Disease (ESRD) requiring hemodialysis (62.7%) and kidney transplants. All patients carried a wide range of comorbidities, including hypertension (70.1%), diabetes (50.7%), cardio-vascular disease (46.2%) and others. The summary of subjects’ characteristics in these two cohorts is shown in **Table 1**. The details of patients and normal control cohort, with respect to demographic characteristics, disease associations and profiles of anti-SARS-CoV-2 IgG antibodies are shown in **Table 2** and **Supplementary Table S2**, respectively. Subjects were genotyped for HLA during clinical evaluation at Columbia University Irving Medical Center (CUIMC) between September 2022 and January 2024 and confirmed to be either homozygous or heterozygous for the HLA-A*02:01 allele.

**Table 1:** Demographic characteristics of kidney patients and normal controls.

Characteristics	Patients	Normal Controls	
		Potential Kidney Donors	Other blood donors
*Cases*	*67*	*51^b^*	*15*
*Ages (years) median (IQR)*	*64.0 (52*–68)	*49.0(37-60)*	*unknown*
Gender			
*Sex F (F%)*	*21(31.4%)*	*34(68%)*	*unknown*
Kidney Disease Stages			
*Advanced CKD III to V (%)*	*30(44.7%)*	*N.D.*	*unknown*
*ESRD*	*34 (50.7%)*	*N.D.*	*unknown*
*Hemodialysis*	*42 (62.7%)*	*N.D.*	
*Acute Kidney injuries (%)*	*9 (13.4%)*	*N.D.*	*unknown*
Comorbidities			
*Hypertension (%)*	*47 (70.1%)*	*N.D.*	*unknown*
*Diabetes mellitus (%)*	*34 (50.7%)*	*N.D.*	*unknown*
*Cardio-vascular diseases (%)*	*31 (46.2%)*	*N.D.*	*unknown*
*Immuno-suppression (previous organ transplants) (%)*	*3 (4.5%)*	*N.D.*	*unknown*
*Other Congestive Diseases (%)*	*2 (3.0%)*	*N.D.*	*unknown*
*unaviable*	*1 (1.5%)*		
HLA-A*02:01 genotypes			
*Homozygous*	*8 (12%)*	*5 (15.6%)*	*1 (6.3%)*
*Heterzygous*	*59 (88%)*	*46 (84.6%)*	*15 (93.7%)*
COVID-19 status^a^			
*Ongoing COVID disease*	*0*	*0*	*0*
*COVID-19 vaccination only (%)*	*20 (29.8%)*	*13 (25.5%)*	*1 (6.3%)*
*SARS-CoV-2 Infection with/without vaccination (%)*	*45 (67.2%)*	*36 (70.6%)*	*14 (93.7%)*

a Estimated from anti-SARS-CoV-2 IgG assay (detailed in **Supplementary Tables S1**; **Table 2**).

b Subject M12829 was removed from the cohort due to lack of anti-SARS-CoV-2 anti-nucleocapsid (N) and anti-Spike I IgG antibodies.

**Table 2:** Summary of characteristics of kidney patients.

Case #	Sample#	Age	Sex	Kidney problem stages	Dialysis	Cardiovascular disease	Diabetes	Hypertention	Autoimmune diseases	Infection	Accute kidney injury	Previous organ transplant (use of immunosupprants)	Tetramer induction by S peptide- (Fold)^b^	SARS-CoV-2 IgG (S1)
1	M07225	53	F	ESRD	1	1		1					2.0	4113
2	M07227	76	M	ESRD		1		1					1.0	24173
3	M07229	66	F	CKD stage 4				1					194.0	22740
4	M07271	75	M	ESRD	1	1	1						1.0	16816
5	M07363	42	M	Acute pyelonephritis without lesion of renal medullary necrosis CKD	1		1	1			1		2.0	22086
6	M07364	47	M	ESRD	1			1					20.0	21551
7	M07415	36	M	ESRD	1			1		LTBI (latent tuberculosis infection)			29.3	18478
8	M07417	68	M	CKD		1	1	1					1.0	13377
9	M07477	43	F	ESRD	1		1	1			1		2.0	N.D.
10	M07586	56	F	ESRD	1	1		1					2.0	19208
11	M08362	63	M	CKD stage IV			1						3.0	18646
12	M08480	26	M	ESRD	1	1		1			1		62.0	15554
13	M08481	21	F	CKD stage 5	1			1					4.0	21409
14	M08527	49	M	ESRD				1					2.8	21128
4.7	M08618	62	M	ESRD	1								4.7	22186
16	M08619	64	M	CKD stage 5	1	1		1		HIV infection, symptomatic			33.6	18195
17	M08620	62	F	ESRD	1	1	1	1					2.0	23107
18	M08621	66	M	CKD stage 5	1	1	1	1					0.1	18644
19	M08739	62	M	ESRD	1	1	1			COVID-19			4.6	19495
20	M08870	70	F	ESRD			1	1					3.0	21106
21	M10354	54	F	ESRD	1		1	1					18.0	14611
22	M10355	59	M	AKI (acute kidney injury) CKD stage 5		1	1	1			1		0.0	17511
23	M10445	64	M	CKD stage 3a									4.1	16325
24	M10448	73	M	CKD			1						13.6	17469
25	M10471	40	M	ESRD	1	1				AIDS			1.1	10597
26	M10492	58	F	ESRD	1	1	1	1					9.9	15757
27	M10500	66	F	ESRD			1	1				Kidney 7/5/07, 8/19/15	1.9	14453
28	m10563	78	M	CKD stage 5	1								1.2	16114
29	m10565	52	M	ESRD	1	1		1					5.0	18728
30	M10585	67	M	CKD stage 5		1	1	1		COVID-19			31.1	2983
31	M10699	67	M	CKD stage IV		1	1	1					27.3	6229
32	M10701	75	F	CKD stage 5		1		1					2.1	13926
33	M10794	75	M	ESRD	1	1	1						15.8	16244
34	M10858	52	F	ESRD	1			1		Currently asymptomatic HIV infection, with history of HIV-related illness			0.9	16225
35	M10869	33	F	CKD stage 4	1				1 (lupus)				16.3	20363
36	M10893	39	M	ESRD	1		1	1		COVID-19			7.2	17549
37	M10914	45	F	ESRD	1		1						4.0	15189
38	m11063	72	M	CKD stage 4		1	1						1.0	10811
39	M11143	66	M	Acute kidney injury superimposed on CKD				1			1		1.8	15359
40	m12069	66	M	CKD stage 4	1		1	1					11.3	4915
41	m12087	68	M	ESRD									12.6	23652
42	m12088	46	F	polycystic kidney disease CKD stage V	1		1						4.5	20021
43	m12114	70	M	CKD, stage V			1	1					1.0	18046
44	m12320	46	M	ESRD	1			1					0.0	19018
45	m12341	77	M	ESRD	1	1	1	1		Bloodstream infection due to central venous catheter			1.7	17742
46	m12597	68	F	CKD stage IV									45.0	23351
47	m12598	27	M	CKD stage 5 ESRD	1			1			1		2.0	15394
48	m12864	61	M	unknown	1								13.5	18721
49	m12877	64	M	CKD		1		1					171.3	17614
50	m12878	63	M	ESRD	1	1		1			1		0.2	8601
51	m13203	72	M	ESRD	1					COVID-19 (11/25/2024)			14.8	14693
52	m13333	55	F	CKD, stage V				1					1.4	12484
53	m13399	68	M	CKD	1	1		1				2012 lung transplant	2.4	16867
54	m13403	72	M	CKD stage IV			1						0.4	15505
55	m13467	30	M	CKD stage IV	1		1	1					33.5	14559
56	m13583	65	M	CKD stage IV		1	1						0.6	19347
57	m13585	72	F	CKD stage IV	1	1	1	1			1		11.6	N.D.
58	m13589	57	M	CKD stage V	1		1				1	Kidney 4/19/2020	41.9	26694
1	M13738	30	F	CKD stage V	1			1					1.0	3373
60	M13802	68	M	CKD stage IV	1	1	1	1		Discitis of lumbar region			29.8	21254
61	M13861	54	M	ESRD, Congenital single kidney1	1	1		1					3.0	24881
62	M13865	72	F	ESRD, CKD stage 4				1					3.3	31439
63	M13880	68	F	ESRD	1	1	1	1		COVID-19 (4/2021)			12.5	23173
64	M13947	67	M	ESRD	1	1		1	1 (systemic sclerosis)	Primary biliary cholangitis with systemic sclerosis			1.0	24610
65	M13987	64	M	ESRD	1	1	1	1					9.3	27318
66	M13391	57	M	ESRD		1	1	1		COVID-19			1.0	19464
67	M13992	77	M	CKD stage 4			1	1		COVID-19			212.0	25670
Median		64											3.3	18046
Q1		52											1.4	15367
Q3		**68**											13.6	21191
Numbers^a^ (counts)			**F=21**	***CKD (III to V) =30, ESRD=34***	42	31	34	47	2		9	3		

a 1 = yes.

b extracted from **Figure 2D**.

c CKD (III to V): patients (counts) with advanced chronic kidney diseases. ESRD: patients (counts) with End-Stage Renal Diseases.

### 3.4 HLA-A2:01/S1_269-277_ (hereafter referred as A2/S_269_) ^+^ CD8^+^ T cells are detectable only in SARS-CoV-2 spike seropositive individuals

To determine whether prior SARS-CoV-2 infection or COVID-19 vaccination is necessary for the detection of A2/S_269_-specific CD8^+^ T cells in peripheral blood, we first pre-screened 11 subjects, selected as naïve controls, for anti-nucleocapsid (N) and anti-S1 IgG antibodies. These individuals had serum samples collected either prior to the COVID-19 pandemic (before January 1, 2020) or during early 2020 when they were found to be negative for COVID-19. As shown in **Figure 2A**, all naïve subjects were tested negative for both anti-S1 and anti-N IgG antibodies.

**Figure 2:**
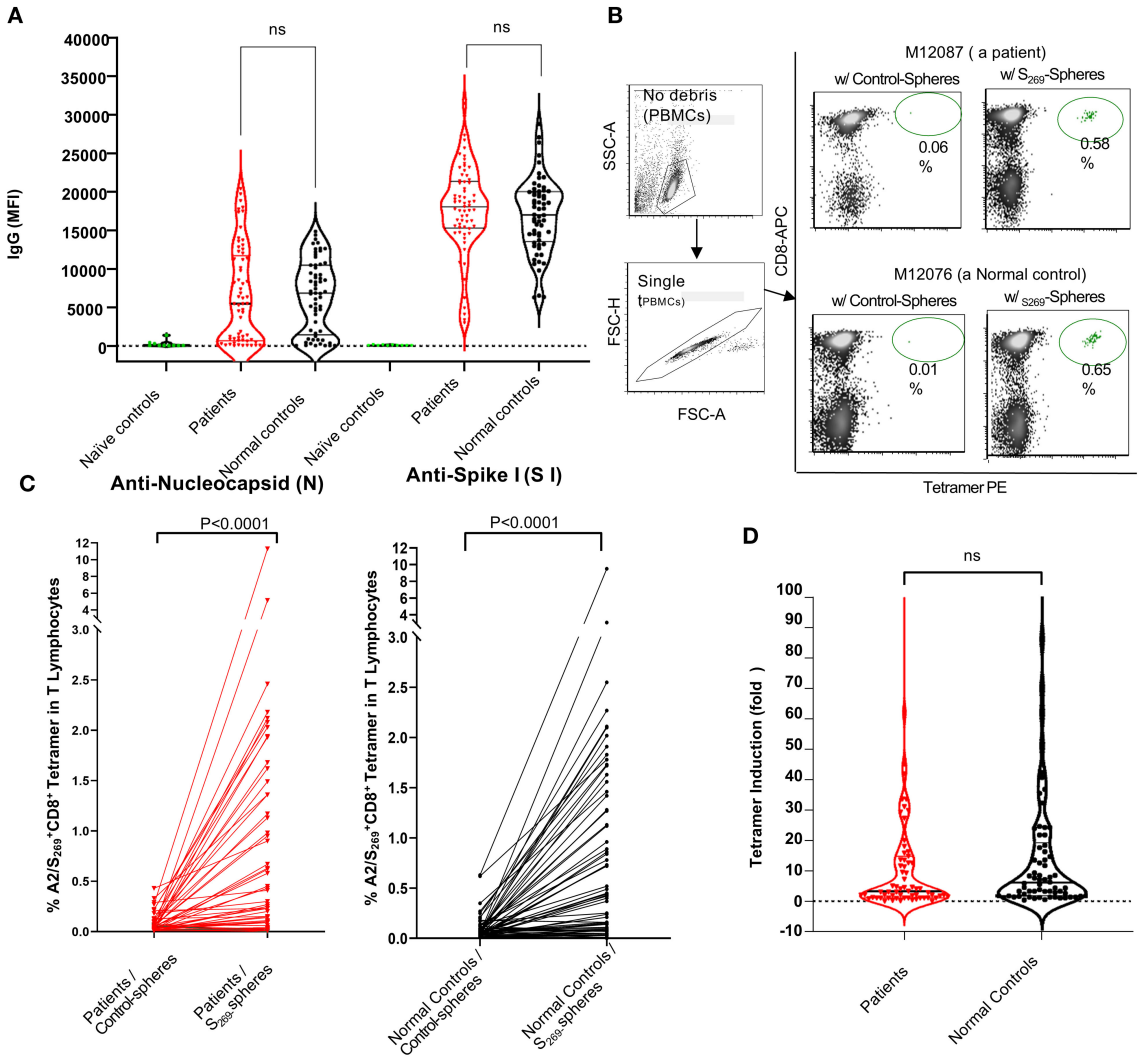
Analyses of anti-SARS-CoV-2 humoral and cellular responses in normal controls and kidney patients. **(A)** Violin Plot to show the levels of anti-SARS-CoV-2 IgG (anti-nucleocapsid and anti-S1) in individuals of naïve controls (n=11), normal control (n=66) and kidney patient individuals (n=67). Note that Naïve controls were designated as individuals whose sera were collected prior to or during 2020 with confirmation negative for COVID-19 tests done on nasal swabs. NS, no significance. B and C: Induction of A2/S_269_^+^CD8^+^ T subset cells in PBMCs by *in vitro* stimulation with either control-(no peptide) spheres or SARS-CoV-2S_269-_-peptide conjugated spheres in normal controls and kidney patients. Representative results from one patient (M12087) and one normal control (M12076) were shown in **(B)** Compiling results from data of all subjects from both cohorts were shown in **(C)** Statistics of results were calculated by Mann-Whitney U (two-tailed) test. *p* < 0.05 was considered statistically significant. **(D)** Violin Plot to show no significant difference in induction of A2/S_269_^+^ CD8^+^T cell subsets between normal controls and patients. Tetramer induction was presented as folds (frequency of tetramer^+^ with peptide conjugated spheres/ frequency of tetramer^+^ with control-spheres). The *p* value returned by Mann-Whitney U (two-tailed) test was 0.3280. N.S, no significance.

In a parallel experiment, unstimulated PBMCs from a subset of these individuals (n=6) were analyzed using A2/S_269_ tetramer staining. None showed detectable (<0.01%) levels of A2/S_269_^+^ CD8^+^ T cells. *In vitro* stimulation with S_269–277_ peptide-conjugated spheres in the presence of cytokines also failed to increase the frequency of these cells (data not shown). These findings suggest that the emergence of A2/S_269_^+^ CD8^+^ T cells only occur after the exposure to SARS-CoV-2 antigens either through natural viral infection or COVID-19 vaccination. For this reason, we recruited only subjects with adequate humoral responses against SARS-CoV-2 for the study of cellular immune response.

### 3.5 Kidney transplant candidates mount adequate humoral responses to SARS-CoV-2

Analysis of anti-SARS-CoV-2 IgG antibodies in all eligible subjects across both cohorts showed no significant differences in humoral immune responses between kidney transplant candidates and normal controls, with respect to the levels of anti-N IgG and anti-S1 IgG (**Figure 2A**). Specifically, levels of anti-Spike S1 IgG antibodies were comparable between patients (median MFI: 18,046) and normal controls (median MFI: 17,016), with statistical analysis by Mann-Whitney’s two-tailed U test yielding *p*=0.2928. Similarly, anti-nucleocapsid (N) IgG levels did not differ significantly between patients (median MFI: 5,438) and controls (median MFI: 6,846); *p*=0.5824.

Given that both natural SARS-CoV-2 infection and COVID-19 vaccination can induce anti-S1 IgG production, while anti-N IgG is produced only following infection, these results indicate that patients with chronic kidney disease awaiting transplantation, like their normal counterparts, can mount equally effective humoral immune responses against SARS-CoV-2 following either COVID-19 vaccination and/or SARS-CoV-2 infection.

### 3.6 Kidney transplant candidates display similar strong A2/S_269_^+^CD8^+^ T cellular responses following *in vitro* stimulation

To evaluate the ability of kidney transplant candidates to mount antigen-specific CD8^+^ T cell responses, we stimulated PBMCs from subjects of both patient and control cohorts with either control-spheres or SARS-CoV-2 S_269–277_ peptide-conjugated spheres and cultured them *in vitro* as described (**Figure 1A**). All samples, arranged in chronological order by DNA ID, were thawed and processed in batch. A2/S_269_-specific CD8^+^ T cell subsets were subsequently detected via A2/S_269_ tetramer staining. A representative flow cytometric analysis of one patient (M12087) and one normal control (M12076) is shown in **Figure 2B**. A summary of analyses from all subjects of both cohorts is shown in **Figure 2C**.

Stimulation with control-spheres yielded low baseline frequencies of A2/S_269_^+^ CD8^+^ T cells: 0.088 ± 0.070% (Mean ± S.D.), stimulation with S_269–277_peptide-conjugated spheres significantly increased the frequency of A2/S_269_^+^CD8^+^ T cells to 0.85 ± 0.80% (Mean ± S.D.) in the control cohort. The induction of tetramer^+^ T cell frequencies by peptide-spheres is highly significant (*p <*0.0001, median:0.050 vs 0.425, *n* =65) by the Mann-Whitney U test. Similarly, stimulation with control-spheres or stimulation with S_269–277_peptide-conjugated spheres yielded the tetramer^+^ T cell frequencies of 0.067 ± 0.057% (Mean ± S.D.) and 0.76 ± 0.87% (Mean ± S.D.) in the patient’s cohort, respectively. The induction of tetramer^+^ T cell frequencies by peptide-spheres is also very significant (*p*<0.0001, median:0.040 vs 0.15, *n* =67) by the same statistical test.

To directly compare the magnitude of induction across cohorts, we calculated the fold-change in tetramer-positive cells. As shown in **Figure 2D**, no significant difference (*p*=0.3280) was observed between the control group (median fold increase=5.8) and the patient group (median fold increase=3.3), suggesting that kidney transplant candidates largely retain the capacity to mount antigen-specific CD8^+^ T cell responses, comparable to healthy individuals.

Notably, approximately one-third of subjects in each group exhibited strong expansion of predominantly tetramer positive CD8^+^T cells in response to peptide stimulation. Specifically, 24 of 65 normal controls (36.9%) and 23 of 67 kidney patients (34.3%) demonstrated at least a 10-fold increase and were classified as high responders (HRs). With HRs, the median fold of increase was 24.0 for the control cohort and 20.0 for the kidney cohort, respectively. In contrast, over half of the subjects in each group showed minimal or no induction, indicating a skewed distribution of T cell responsiveness within the population. These data, along with comparable SARS-CoV-2 IgG responses, demonstrate that patients with chronic kidney disease awaiting transplantation can mount robust humoral and cellular immune responses to SARS-CoV-2, despite their underlying comorbidities.

Due to the heterogeneity of the patient cohort with respect to underlying cause of kidney failure, we investigated whether the induction (fold) of A2/S_269_ tetramer^+^ cells was related to various kidney diseases. The induction of antigen-specific T cells was found to be randomly distributed among kidney patients ((**Table 2**). To clarify the roles of these disorders, we categorized kidney patients into two groups: ESRD, comorbidities due to non-immunological causes(hypertension, diabetes, cardio/vascular and acute injury) and ESRD caused by immunological conditions and(including autoimmune diseases and use of immunosuppressants for previous transplant. (**Supplementary Figure S1** demonstrates that all two groups exhibited a similar median fold of induction (3.0) along with comparable interquartile ranges (IQR). A comparison analysis revealed that the differences were statistically insignificant (*p*=08157). The analyses indicated that comorbidities, immunosuppression, and autoimmune status did not influence the induction of tetramer-positive T cells or the responses specific to peptide antigens.

Both the patient and normal control groups exhibited comparable frequencies of high and low responders, suggesting that variability is not limited to disease status. Factors such as HLA genotype, age, sex, and comorbidities did not significantly influence the strength of the response. Furthermore, the capacity to respond to peptide antigen-specific T cell stimulation was found to be independent of the levels of anti-S1 SARS-CoV2 IgG, as every individual within the cohorts demonstrated adequate humoral responses.

### 3.7 A2/S_269_^+^ CD8^+^ T cell expansion correlates with increased production of IFN-γ and TNF-α in both normal controls and kidney patients

Pro-inflammatory cytokine secretion, particularly IFN-γ and TNF-α, is a hallmark of cytotoxic CD8^+^ T cell function and is essential for effective immunity against intracellular pathogens and tumor cells (29). To investigate whether antigen-specific A2/S_269_^+^CD8^+^ T cells can produce inflammatory cytokines, we first performed IFN-γ ELISpot assays on PBMCs from two A2/S_269_^+^ normal donors, M10315 and M11116, following *in vitro* stimulation. PBMCs were cultured with peptide-conjugated spheres targeting SARS-CoV-2 S_269–277_, SARS-CoV-2 ORF1ab (2332–2340) (19), or CMVpp65 (495–504) (30), along with cytokines as previously described. Post-stimulation, cells were split into two parts: one was analyzed for A2/tetramer^+^ frequencies, and the other was cultured for an additional 16 hours in serum-free medium with cognate peptides or PHA (as a positive control) to measure the frequency of IFN-γ secreting cells.

**Figure 3:**
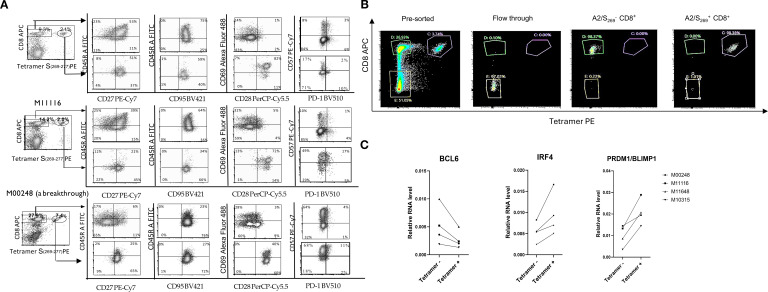
A2/S_269_^+^CD8^+^T cells exhibited effector-memory T cell phenotype. **(A)** Decreased expression of naïve and central memory marker CD45RA and increased expression of effector markers, CD57 in A2/S_269_^+^ T cell subsets when compared to bystander CD8^+^ T cells. PBMCs from 3 normal controls were analyzed for various memory T cells markers (CD45RA, CD27, CD95), effector markers (PD-1 and CD57) and co-stimulatory markers CD28 by Flow Cytometry. The histogram analysis of these results is shown in **Supplementary Figure S2**. Results were shown as Mean ± S.D. **(B)** A representative of sorting profile of A2/S_269_^+^ CD8^+^ T cell subset and bystander CD8^+^ T cells; typically, >97.5% purities. **(C)** Down-regulation of BCL6 and up-regulation of PRDM/Blimp1and IRF4 in A2/S_269_^+^CD8^+^ T cells, compared to bystander CD8^+^ T cells. RNA from 4 Tetramer A2/S_269_+ subsets and 4 tetramer- subsets were analyzed by quantitative RT-PCR with endogenous GAPDH as an internal control. The gene expression relative to housekeeping gene GAPDH was calculated using the formula 2^-^ *^ddCt^*, where *ddCt* =(*Ct*[gene]-*Ct*[GAPDH]) and *Ct* is the crossing threshold value returned by the PCR instrument for every gene amplification. The results were recorded as an average from duplicate.

Tetramer staining revealed robust induction of A2/S_269_^+^CD8^+^ T cells and HLA-A*02:01/CMVpp65_495-503_ (hereafter referred to as CMVpp65_495_)^+^ CD8^+^ T cells following cognate peptide stimulation, with 10- and 7-fold increases, respectively (**Figure 3A**; **Supplementary Figure S4**). In contrast, A2/ORF1ab_2332_-specific T cells were undetectable by tetramer staining or cytokine release, suggesting that this epitope may be cryptic or poorly immunogenic. Consistent with the tetramer results, ELISpot assays showed negligible IFN-γ producing cells from unstimulated or control-sphere-treated PBMCs. In contrast, upon re-challenge with free cognate peptides, a marked increase, often >100-fold IFN-γ producing cells, was observed in cultures stimulated with A2/CMVpp65_495_ peptide-spheres, which served as ELISpot positive control, and cells stimulated with A2/S_269_ peptide-spheres.

**Figure 4:**
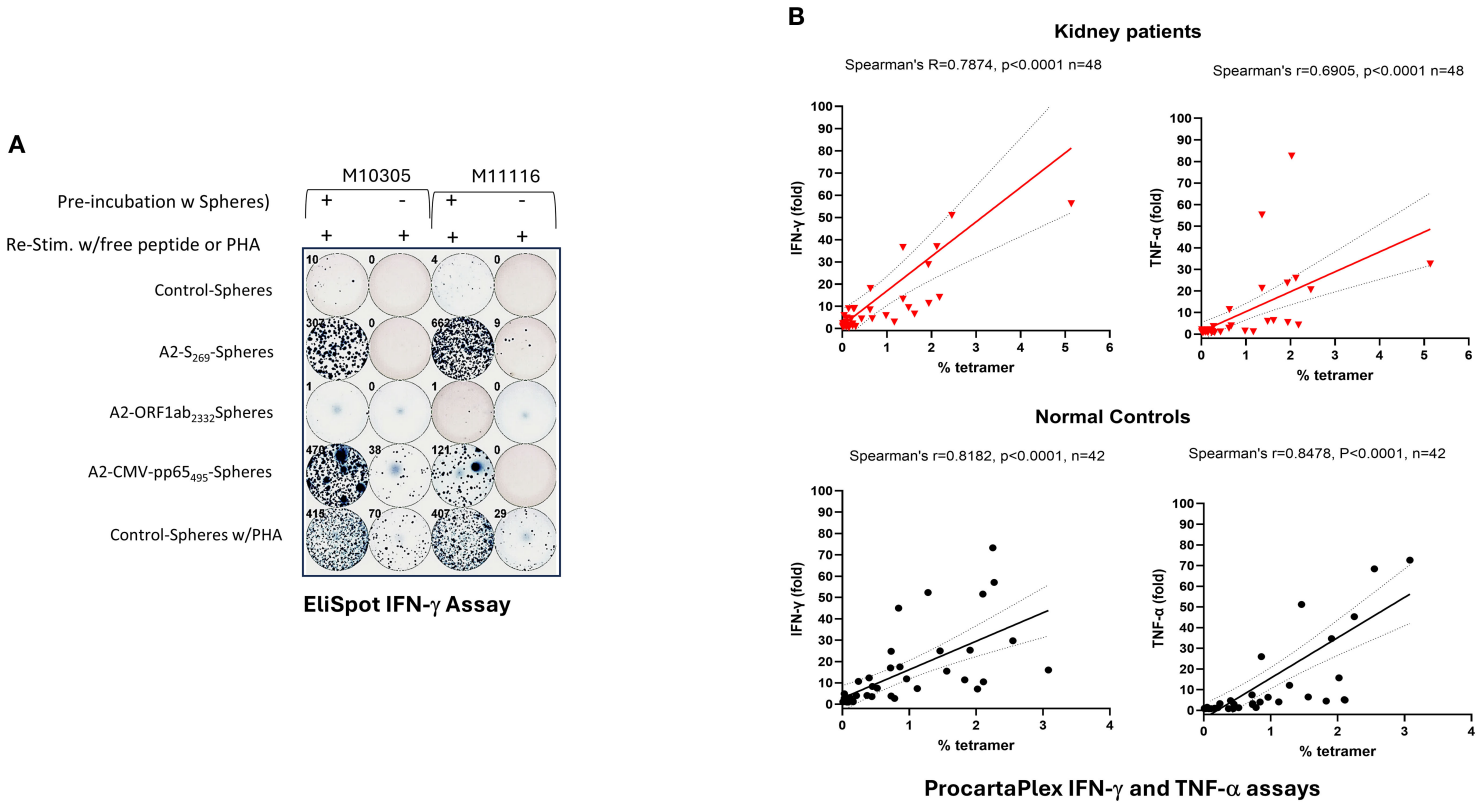
Induction of IFN-γ and TNF−α by PBMCs stimulated with A2/S_269-_ peptide conjugated spheres in normal controls and kidney patients. **(A)** Increased IFN-γ producing cells in PBMCs by *in vitro* stimulation with various peptide conjugated spheres. In the ELISPOT assay, Cells cultured with A2-CMV-pp65_495_-spheres or with PHA (0.2x)were used as the positive controls, whereas cells cultured with control-spheres or without *in vitro* stimulation were used as the negative controls, respecitvely. Numbers of IFN-γ producing cells were visualized and automatically calculated by ImmunoSPOT2™ software. **(B)** ProcartaPlex assays of secreted IFN-γ and TNF-α in supernatants from cells stimulated with SARS-CoV-2S_269-_peptide conjugated spheres. Spearman’s rank correlation and simple linear regression were used to determine the relationship between the frequency of tetramer^+^ vs the fold of cytokine (IFN-γ and TNF-α) release by cognate peptide stimulation. .

To further evaluate the relationship between antigen-specific CD8^+^ T cell expansion and cytokine release in all subjects, we employed Luminex-based multiplex cytokine assays (ProcartaPlex), allowing us to measure the release of several cytokines (IFN-γ, TNF-α and MIP-1β) simultaneously. PBMCs from both normal controls and patients’ cohorts were stimulated *in vitro* with S_269–277_ peptide-Spheres as previously described, then re-stimulated with cognate peptide. Cytokine levels released in supernatants were measured as fold induction (with peptide/without peptide), and results were plotted against A2/S_269_^+^ tetramer frequencies. As shown in **Figure 4B**, strong positive correlations were observed between A2/S_269_^+^ CD8^+^ T cell frequencies and cytokine production across both cohorts: In normal controls (n=42): IFN-γ: Spearman’s *r*=0.8182, *p*<0.0001; TNF-α: Spearman’s *r*=0.8478, *p*<0.0001. In kidney patients (n=48): IFN-γ: *r*=0.7874; *p*<0.0001. TNF-α: *r*=0.6905, *p*<0.0001. No significant correlation was observed between A2/S_269_^+^ CD8^+^ T cell frequency and MIP-1β production (data not shown), suggesting a biassed pro-inflammatory cytokine profile.

Our findings indicate that *in vitro* peptide-sphere stimulation of PBMCs leads to clonal expansion and functional differentiation of A2/S_269_-specific CD8^+^ T cells into IFN-γ and TNF-α producing effector cells. Importantly, PBMCs from kidney transplant candidates exhibited expansion and cytokine-producing capacities comparable to those of healthy controls, highlighting their preserved potential to mount effective cellular immune responses despite their underlying comorbidities.

### 3.8 HLA-A2/S_269_^+^ CD8^+^ T Cells display both memory and effector T cell surface markers

The substantial enrichment of SARS-CoV-2 antigen-specific CD8^+^ T cells in our system enabled a direct comparison of phenotypic differences between T cells expanded by cytokines alone (bystander tetramer negative CD8^+^ T cells in the same culture) and those expanded by both cytokines and peptide antigen (tetramer positive CD8^+^ T cells). *In vitro* induced A2/S_269_ antigen specific T cells from three HLA-A*02:01 normal controls, in addition to M10315 and M11116, also including donor M00248, who had recently recovered from a breakthrough SARS-CoV-2 infection, were analyzed for expression of canonical memory and effector T cell surface markers via flow cytometry.

The memory T cell markers were evaluated including: CD27, CD45RA, CD95, and CD69 (a tissue-resident memory T cell marker), effector and activation markers including CD57, PD-1, and the co-stimulatory molecule CD28. As shown in **Figure 3A** and **Supplementary Figure S2**, the tetramer^-^CD8^+^ T cell population from 3 peptide-spheres/cytokine-stimulated cultures exhibited the following phenotype: CD45RA^+^(54 ± 20%, MFI: 2235 ± 709), CD27^+^ (45 ± 18%, MFI: 1878 ± 1164), CD28^-^(8 ± 3%, MFI:78 ± 53), CD69^iow^(27 ± 8%, MFI:1032 ± 208), CD95^+^(100%,MFI: 4301 ± 678), CD57^low^ (30 ± 25%, MFI: 1770 ± 1951) and PD-1^-^(6 ± 1.7%, MFI: 779 ± 184). These phenotypes were indistinguishable from CD8^+^ T cells stimulated with control-spheres/cytokines (data not shown), indicating naïve/central memory T cell phenotype with minimal antigen-specific activation. In contrast, tetramer^+^CD8^+^T cells (antigen-specific) exhibited a distinct profile: CD45RA^low^(40 ± 12%,MFI:1195 ± 552), CD27^+^(81 ± 10%,MFI:789 ± 594), CD28^+^(93 ± 5%, MFI:10417 ± 890), CD69^+^(71 ± 21%, MFI: 6186 ± 3217), CD95^+^ (99%, MFI: 3139 ± 219), CD57^+^ (55 ± 24%, MFI: 8904 ± 4298) and PD-1^-^(16 ± 4%, MFI: 1081 ± 76).

Comparison of the population of tetramer positive CD8^+^ T cells with bystanders of tetramer negative CD8^+^ T cells, shows that the former has a lower expression of CD45RA (CD45RA^low^) and higher expression of CD57(CD57^bright^), suggesting that tetramer positive, antigen-specific CD8^+^ T cells have differentiated into more mature T cells with effector-memory T cell phenotype during *in vitro* peptide-sphere stimulation. This phenotype of CD45RA^low^CD57^bright^ was also observed in tetramer negative bystander CD8^+^ T cells in the breakthrough individual (M00248).

Importantly, increased expression of CD28 and lack of expression of PD-1 reinforce the interpretation that these cells were not terminally exhausted but rather maintained functional capacity and proliferative potential. Taken together, these results indicate that *in vitro* stimulation with S_269-277-_peptides- conjugated spheres had triggered differentiation of SARS-CoV-2-specific CD8^+^ T cells with highly differentiated effector-memory phenotypes.

### 3.9 Increased expression of IRF4 and PRDM1/BLIMP1 and decreased expression of BCL6 in A2/S_269_^+^CD8^+^ T cells

BCL6 and PRDM1/BLIMP1 are well-established transcriptional repressors (31, 32) originally identified for their roles in B cell differentiation. More recently, these two factors have been recognized as key antagonistic, or “Yin-Yang,” regulators of T cell fate, particularly involved in the differentiation of memory T lymphocytes into effector subsets (33, 34). Another key transcription factor that tightly regulated by both BCL6 and PRDM1/BLIMP1 is Interferon-Regulatory Factor 4 (IRF4). As the promoter of IRF4 contains binding sites for both BCL6 and Blimp-1 (35), IRF4 has been shown to be required for the generation of protective effector CD8^+^ T cells against intracellular bacterium Listeria monocytogenes in a murine model (36). To explore how these transcription factors function in human SARS-CoV-2-specific CD8^+^ T cells, we first sorted cells from *in vitro* S_269-277-_peptide-spheres stimulated PBMCs by Flow Cytometry into tetramer-positive (SARS-CoV-2-specific) and tetramer-negative subsets (bystander). A representative graph (**Figure 3B**) representing results obtained after sorting showed the purity of CD8^+^ T cells was greater than 97.5%. Four tetramer-positive subsets and four tetramer-negative subsets sorted from normal controls were subsequently assayed for RNA expression of BCL6, PRDM1/BLIMP1 and IRF4 by Real-time PCR.

As shown in **Figure 3C**, although heterogeneous, BCL6 RNA was down-regulated by an average of two-fold in all 4 sets of tetramer-positive cells when compared to the bystander tetramer-negative cells. Conversely, IRF4 and PRDM1/BLIMP1 were up-regulated in all 4 sets of tetramer-positive cells, when compared to the bystander tetramer-negative cells, with a similar 2-fold increase. It appears that the transcriptional profile of antigen specific CD8 T cells, characterized by upregulated PRDM1/BLIMP1 and IRF4 differs from that of noncommitted bystander CD8^+^T cells that show BCL6 upregulation. It also supports the notion that PRDM1/BLIMP1modulates transcriptional programs of CD8^+^ T cells during chronic viral infection as indicated in a murine model (37).

### 3.10 A2/S_269_^+^ CD8^+^ T cells exhibit potent antigen-specific cytolytic activity

CD57 (Leu-7) was regarded as a marker of senescence in CD8^+^ T cells (38). However, recent studies indicated that CD57^+^ T cells were not only able to proliferate *in vivo* (39) but also displayed heightened cytolytic potential, defining subsets enriched in lytic granules within both CD8^+^ T cells and natural killer (NK) cells (40, 41). Given that secretion of antiviral cytokines such as IFN-γ and TNF-α (**Figure 4**) does not always correlate with cytotoxic function, as observed in some HIV-specific CD8^+^ T cell responses (42), we next investigated whether A2/S_269_^+^ CD8^+^ T cells exhibit direct cytolytic effects to target cells.

**Figure 5:**
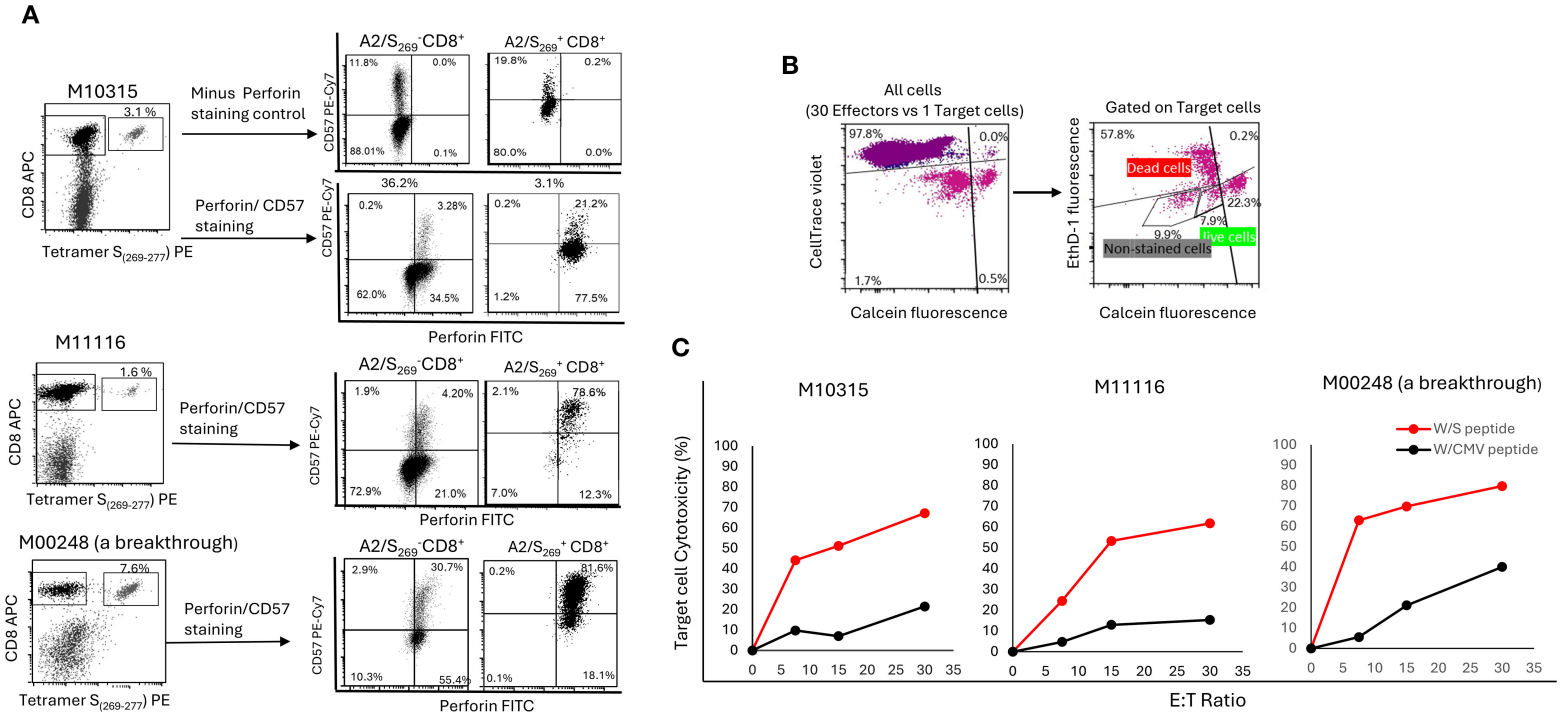
A2/S_269_^+^ CD8^+^T cells were enriched in CD57^bright^/Perforin^high^ subset and were highly cytotoxic to HLA-A*02:01 target cells in an antigen specific- and a dose dependent- manner. **(A)** PBMC from 3 HR normal controls were *in vitro* stimulated as described. After tetramer staining, cells were counter-stained with CD57 and perforin mAbs. Minus-Perforin staining was also performed in M10315 to rule out some false positivity associated with intracellular staining of perforin. The histogram of this analysis is shown in **Supplementary Figure S2**. **(B)** Gating strategy for cell mediated cytotoxicity. Effectors which stained with CellTace Violet were first to be gated out, followed by gating out unstained target cells. Calcein AM (green) positive target cells were counted as live cells, whereas double positive [Ethidium (red) and Calcein AM (green)] and Ethidium positive (red) target cells were all counted as dead cells. **(C)** Target specific and dose dependent of A2/S_269_^+^CD8^+^ T cell mediated cytotoxicity. The percentage of effector cell mediated cytotoxicity was calculated using the following equation: Cytotoxicity (%) = 100*(total deaths target cells-spontaneous deaths) / (100-spontaneous deaths).

We first determined the co-expression of CD57 and perforin within tetramer^+^ CD8^+^ T cells. As shown in **Figure 5A** and **Supplementary Figure S3**, three PBMCs from healthy controls, including a breakthrough donor M00248, showed high co-expression of CD57 expression and intracellular perforin expression within the tetramer^+^ subset. On average, 60.5% ± 33% of tetramer^+^ cells were double-positive for CD57^bright^ and perforin^+^. In contrast, CD57^dim^/tetramer^-^cells were largely perforin-negative (80.5% ± 5.5%). This result is consistent with the finding by Chiang et al. (40) that CD57^bright^ marks perforin-expressing CD8^+^T cell subsets. Notably, normal control M10248, who had a recent breakthrough COVID-19 infection, exhibited elevated perforin expression across both tetramer^+^ and tetramer^-^ subsets, potentially reflecting immune stimulation following recurrent SARS-CoV-2 infections. Nonetheless, histogram (**Supplementary Figure S3**) analysis confirmed significantly higher perforin expression in tetramer^+^ cells (MFI=11,188) compared to tetramer^-^ cells (MFI=3,337) in this donor.

To evaluate cytolytic function directly, we conducted a cell-mediated cytotoxicity assay using HLA-A*02:01-expressing T2 cells pulsed with either cognate SARS-CoV-2 S_269–277_ peptide or with irrelevant peptide CMVpp65_495–503_ as a negative target control. Effector cells, generated by PBMCs primed with S_269–277_ peptide conjugated spheres, were co-cultured with cognate S peptide loaded target T2 cells or irrelevant CMV peptide loaded target T2 cells at varying effector-to-target (E: T) ratios. As shown in **Figure 5B**, peptide-primed effectors demonstrated the strongest dose-dependent cytotoxicity, lysing 62.8%, (Range: 51.2-69.8%) of cognate target cells at an E:T ratio of 15:1. In contrast, target cells loaded with CMV peptide were lysed in a much lower percentage (Mean=13.4%, Range: 7.0 - 21.3%) at the same E:T ratio. This result demonstrates that cell-mediated cytotoxicity was strictly antigen-specific, as less killing was observed when targets were loaded with a potentially irrelevant CMV-derived peptide.

Of note, antigen specific target cell killing was well correlated with the frequency of A2/S_269_^+^ T cells (**Figure 5A**). Donor M10248 (a breakthrough) with 7.6% tetramer^+^ T cells killed 63% of targets. Donor M10315 with 3.1% tetramer^+^ T cells killed 44.2% of targets, donor M11116 with 1.6% tetramer^+^ T cells killed 24.5% of targets, all had an E:T ratio of 7.5:1.

Taken together, our findings show that priming with SARS-CoV-2 S_266–274_-conjugated spheres efficiently generates a distinct population of antigen-specific CD8^+^ T cells characterized by high expression of both CD57 and perforin, key markers associated with cytotoxic effector function, as shown before (40). These CD57^bright^/perforin^high^ T cells exhibited potent antigen-specific killing of peptide-loaded target cells in a dose-dependent and HLA-restricted manner, underscoring their robust functional cytolytic potential. In conclusion, A2/S_269_^+^ CD8^+^ T cells are not only capable of antigen-specific proliferation and cytokine secretion but also possess strong cytolytic potential. Their perforin expression and robust killing of peptide-loaded targets highlight their functional effector capacity, suggesting their suitability for T cell-based antiviral therapies.

## 4 Discussion

### 4.1 A2/S_269_+ antigen-specific CD8^+^ T cells as SARS-CoV-2-specific cytolytic effector cells in T cell therapy

In this study, we show that *in vitro* stimulation of T cells with peptide coated microspheres, memory A2/S_269_+ cells can be rapidly expanded and differentiated *in vitro* into T cells with an effector-memory and cytotoxic phenotype and function. These cells exhibit potent cytolytic activity and unique expression of surface CD28 and CD57 and of effector -related -transcriptional factor PRDM1/BLIMP1 and IRF4. Multiple rounds of division have evidently not resulted in exhaustion since these T cells remain CD28 +. To our knowledge, our current findings describe for the first time the distinct phenotype of these antigen specific cytotoxic CD 8 T cells.

Although initially considered indicative of sub-optimally Cov-2 activated T cells, in comparison to T cells activated by other viral epitopes (e.g., EBV A2/BMLF_1280_) (14)), longitudinal studies of cellular immune reactivity have subsequently revealed that a subset of SARS-CoV-2 specific T cells, including A2/S_269_-specific CD8^+^ T cells, persist for long periods of time(1–2 years). This was found both after “short” and “long “COVID-19 disease, reflecting the expansion of SARS-CoV-2 T memory cells specific for unique epitope(s) with stringent requirements of fitting TCR configuration (15). In particular, T cell receptor (TCR) analyses have identified a biased TCR repertoires specific to A2/S_269_, commonly featuring TRAV12-1 α chain gene usage (16). Hence, a restricted T cell repertoire, probably directed toward an immunodominant viral epitope, seems to be conserved in different populations.

The frequencies of A2/S_269_-specific CD8^+^T cells drastically increased (>10-fold up to 200-fold) and reached approximately 0.5%-3% of total CD8^+^ T cells in approximately 35% of individuals. Given that CD8 T cells increase from one third of total circulating T cells to 50-60% after peptide-sphere stimulation in 14-day cultures, we estimate that S-antigen-specific CD8^+^ T cells may have been enriched by more than 50-fold during the *in vitro* experiment. This finding highlights the therapeutic potential of our study, making it a promising candidate for adoptive T cell therapy.

### 4.2 Lessons from the COVID-19 pandemic: advancing the generation of viral-specific cytotoxic T cells beyond SARS-CoV-2

While the interest in adoptive transfer of SARS-CoV-2-specific T cells has decreased following the approval of antiviral agents such as Paxlovid, the demand for effective viral-specific T cell (VST) therapies remains high, particularly for immunocompromised patients undergoing solid organ transplantation (SOT) or hematopoietic stem cell transplantation (HSCT).

*in vitro* priming of PBMCs with CMVpp65 peptide-conjugated spheres, used as a control for our study, also results in significantly enhanced T cell proliferation (7-fold increase, **Supplementary Figure S4**) and IFN-γ secretion (>100-fold increase, **Figure 4A**). Hence, our approach may be appliable for the generation of VST against CMV, with stronger capacity to elicit both activation and subsequent differentiation. The ex vivo priming and re-infusion of autologous VST does not complicate the immunological conditions of a transplant patient. Unlike third-party T cells, the recipient’s autologous VST cells would not be targets of rejection, ensuring their longevity after transfusion and persistence of anti-viral immunity. Secondly, autologous VST cannot be effectors of graft vs host disease, increasing their safety profile.

Moreover, it is conceivable that in tumors with malignancies carrying well-identified immunogenic epitopes, the peptide antigen coated beads could provide an excellent tool for increasing the immunogenicity of tumor antigens. The method that we have developed, along with other optimizing approaches, such as better peptide design and delivery adjuvants, should facilitate the development of peptide-based vaccines as viable cancer therapeutics (43).

Despite the encouraging potential of antigen-specific T cells for both antiviral and anti-tumor therapies, developing long-term expansion protocols while consistently preserving antigen specificity, continues to be a challenge. Numerous studies have sought to tackle these challenges through various methods, including the complementary use of anti-CD3/autologous feeder cells (44). The implementation of antigen scaffolds constructed on a streptavidin-conjugated dextran backbone with both HLA-restricted peptides and biotinylated CD86 attached (45), or the utilization of recombinant molecules such as T-CEP (46) may be other promising approaches.

Furthermore, single-epitope vaccines may be more susceptible to immunological evasion (47), as mutations or substitutions of this epitope have been observed in the SARS-CoV-2 genome. For this reason, the combination of several well-characterized immunodominant epitopes for initial priming, may be advantageous for generating multiple distinct antigen-specific cytotoxic T cell populations. In the realm of CMV T-cell therapy, the use of pp65 and IE-1 peptides for inducing CMV-specific cytotoxic T cells has yielded favorable outcomes (48, 49).

### 4.3 Limitations of the study

Our study has several limitations that should be considered: 1) Sample size was relatively small due to the limited volume of leftover clinical blood samples (typically 2–4 milliliters),available phenotypic and functional analysis. 2) Sphere efficiency and priming could be eventually optimized by using spheres with magnetic properties which facilitate their retrieval after T cell expansion.3) Biodegradable artificial antigen-presenting cells, such as hyaluronic acid (HA) hydrogen-based platforms, have shown effectiveness in activating tissue-residual memory CD8^+^ T cells *ex vivo* for *in vivo* delivery (50) and may be advantageous also in context of our aims. 4) The small sample size in our study indicates the need for validation in a larger, more diverse cohort.

## Data Availability

Due to ethical/privacy/consent restrictions, raw data will be made available by the corresponding author only upon request.
