# Supplementary material for: Development of potent HLA-A02:01-restricted peptide-based cytotoxic T-cells against SARS-CoV-2 infections in patients awaiting a kidney transplant
Source: Front Immunol. 2025 Oct 6;16:1664371. doi: 10.3389/fimmu.2025.1664371 (PMC12535972; doi:10.3389/fimmu.2025.1664371)
Supplement: Supplementary file 1 [file DataSheet1.pdf]

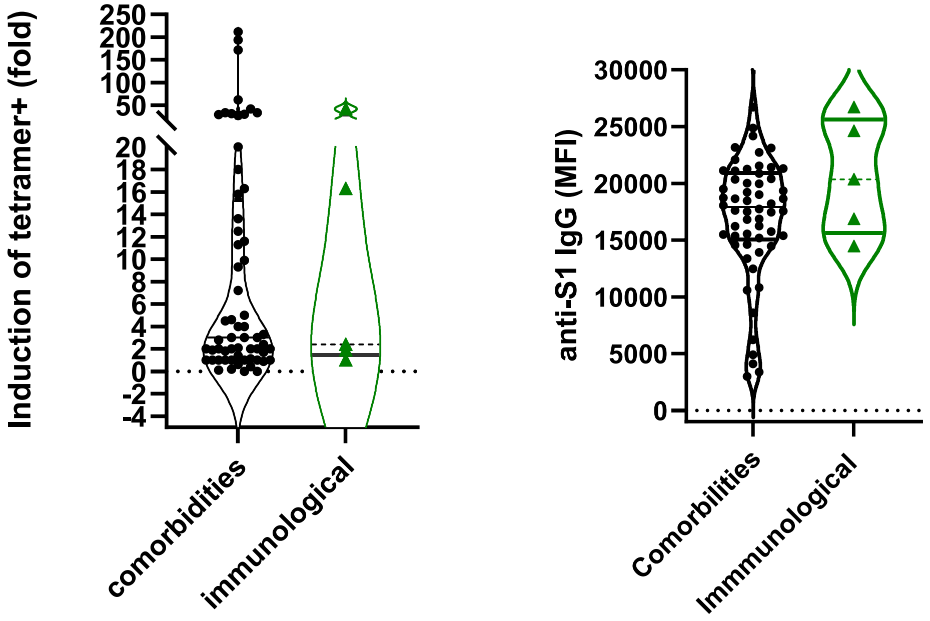

Figure S1: Mann-Whitney tests were performed on 2 subgroups of kidney patients. For comorbidities, n=60, for immunological(autoimmune diseases and the use of immunosuppressants), n=5; p=0.8157 for the comparison of A2/S_{269} tetramer^{+} T cell induction and p=0.2914 for the comparison of anti-S1 IgG levels, respectively. p<0.05 is considered statistically significant. The data was extracted from **Table 2**.

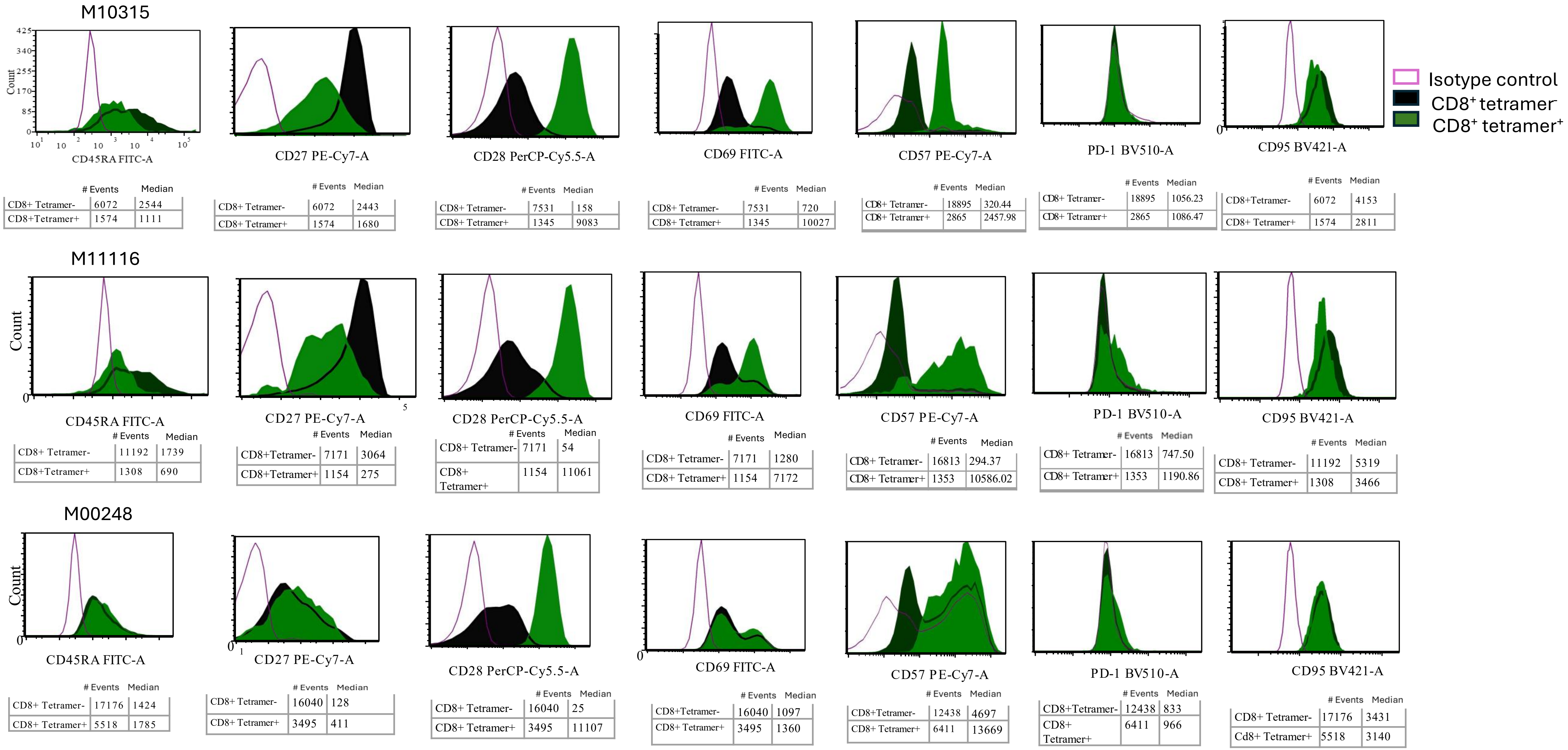

Figure S2: Histogram showed 3 HR PBMCs stimulated with A2S$_{269}$-spheres were stained with surface markers CD27,CD28, CD45RA, CD57, CD69,CD95 and PD-1 as described in **Figure 4A**.

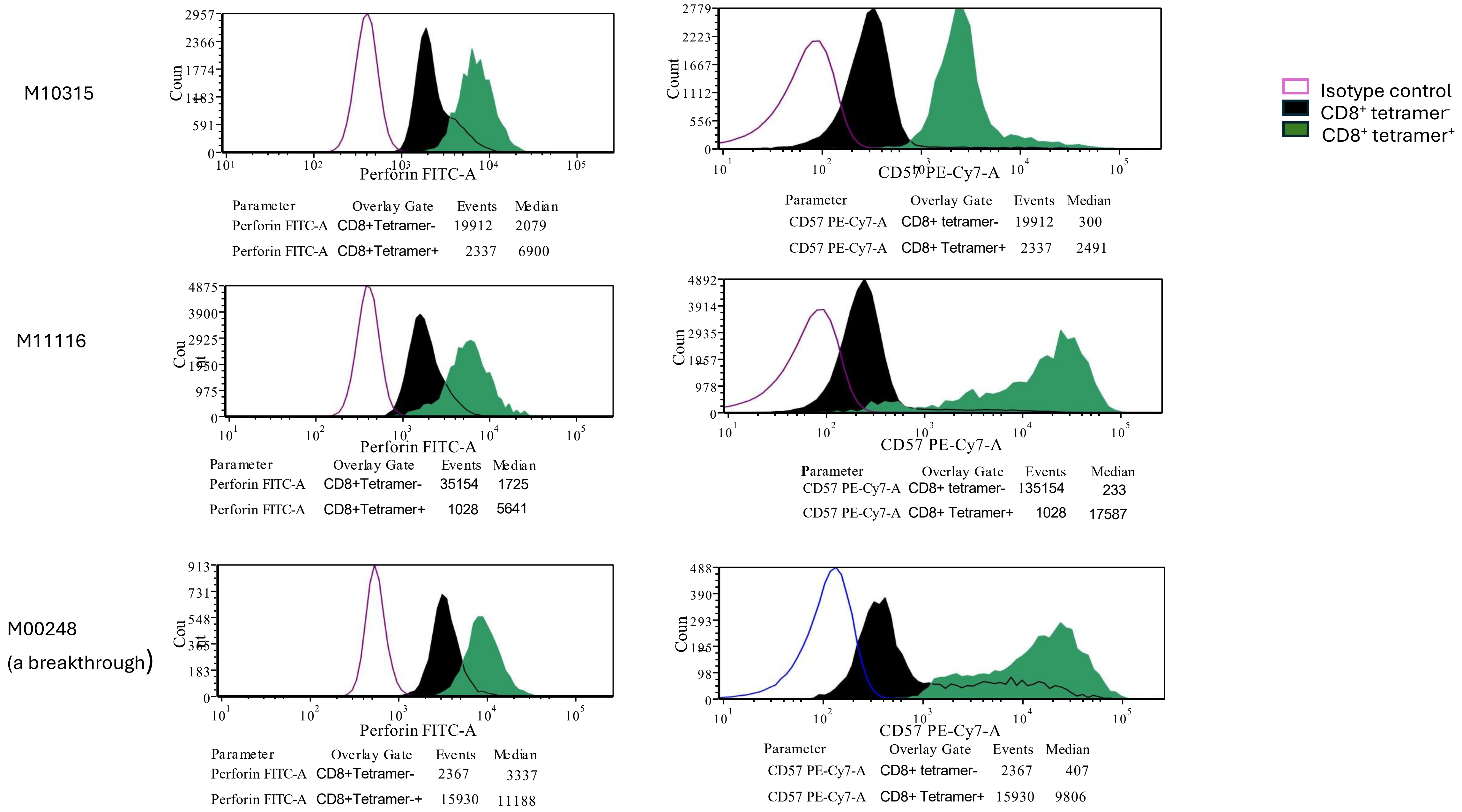

Figure S3: Histogram showed cells stimulated with $A2S_{269}$-spheres were stained with CD57 and perforin as described in Figure **5A**.

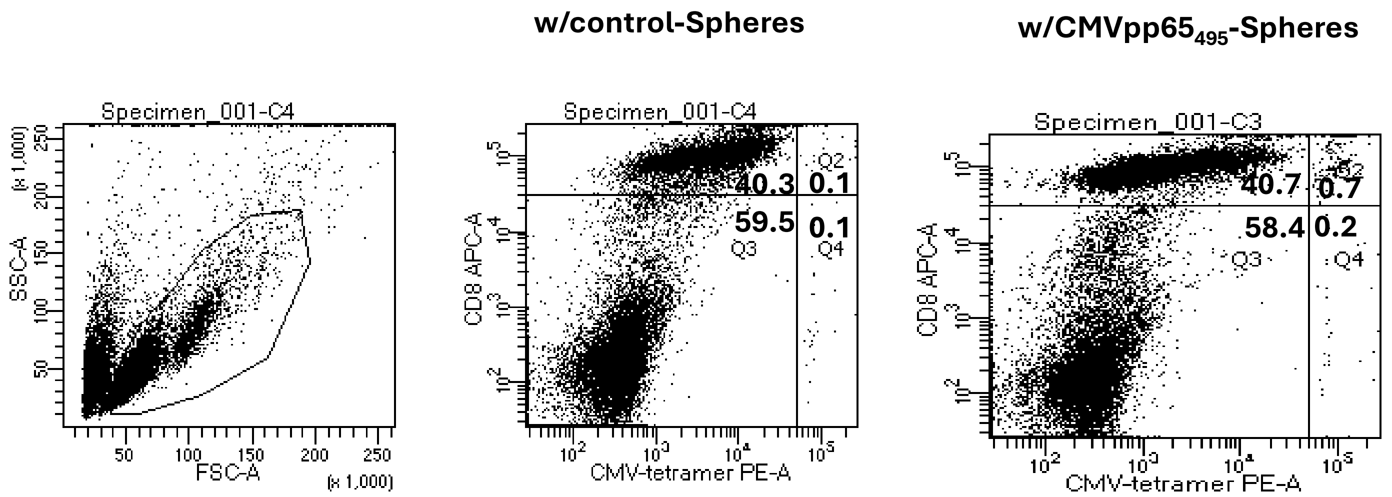

Figure S4: Induction of CMVpp65$_{495}$$^{+}$CD8^{+} T cell subset by *in vitro* stimulation of PBMCs (M10315) with CMVpp65$_{495-}$peptide conjugated spheres and cytokines. The induction of IFN-γ from these cells was shown in **Figure 3A**

Table S1: Detailed Demographic Characteristics of Normal Controls

Potential Kidney Donors

Case #	Sample #	Age	Sex	HLA A locus	anti-Nucleocapsid	anti-S1 IgG	anti-RBD Ig	anti-S2 IgG	previous infection w /wo vaccination	Vaccinatio nly
1	M08429	48	F	A 02:01, 03:01	39	20042	22513.75	11487		**1**
2	M08484	62	M	A 02:01, 03:01	12175	18550.5	16998.75	7108.5	1	
3	M08501	60	F	A 02:01	7025	9785	12315.75	7424	1	
4	M08535	45	F	A 02:01, 03:01	292.5	21800	20857.75	9480.5		1
5	M08537	53	F	A 02:01, 36:01	12646	19731.5	15711.25	16870.5	1	
6	M08557	59	F	A 02:01	253.5	12948	15590.75	9149		1
7	M08558	69	M	A 02:01, 02:05	1256.5	21070.5	19104.75	10523	1	
8	M08737	37	F	A 02:01, 03:01	14861	16962	13134	14616	1	
9	M08782	39	M	A 02:01, 32:01	8280	11795	15920.75	8554.5	1	
10	M08783	37	F	A02:01,69:01	8731	19438	19524.75	9970.5	1	
11	M08788	54	F	A02:01, 26:01	14981	18334	19360.25	7219.5	1	
12	M08816	50	F	A 02:01, 14:BMD	1994	13101	12415	7283.5	1	
13	M10421	30	F	A02:01, 66:01	4731.5	15728.5	14810	7025	1	
14	M10460	52	M	A01:01, A 02:01	793	18811	17032.5	8786		1
15	M10461	62	F	A02:01, A24:02	11525.5	6337	6570	5480.5	1	
16	M10464	49	F	A02:01, A02:01	774.5	10685	12183	8801.5		1
17	M10515	34	F	A 02:01, 68:02	42	17797.5	18417	8433		1
18	M10516	80	F	A 02:01, 29:02	7915.5	14496	12068	9373	1	
19	M10532	60	F	A 02:01, 32:01	6303	13205	13607	7720.5	1	
20	M10538	20	M	A02:01, 31:01	10466.5	13688	11823	11557	1	
21	M10577	42	F	A 02:01, 25:01	7083	12658.5	13226	10268	1	
22	M10580	26	M	A 02:01, 66:01	6846	13361	12625	5076	1	
23	M10595	60	M	A 02:01, 03:01	31	20145	19260	9439		1
24	M10733	62	F	A01:01, 02:01	40	20065	17717	8554		1
25	M10737	63	F	A 02:01, 03:01	3088.5	19961.5	16883	13627.5	1	
26	M10739	47	M	A 02:01, 25:01	9437	18414	13762	11970	1	
27	M10811	56	F	A 02:01, 25:01	5445.5	20799.5	15971	14759	1	
28	M10818	63	M	A 02:01, 32:01	10584	16456.5	13435	11955.5	`	
29	M10909	63	F	A 02:01, 23:CJT	6789	14932.5	12043.5	8613	1	
30	M10910	47	F	A 02:01, 02:02	430	17748	17219	6354		1
31	m11065	48	F	A 02:1, 02:01	43	14514	14908.5	4284		1
32	m11066	53	M	A 01:01, 02:01	49.5	16159	13672.5	8524		1
33	M11285	69	F	A02:01, 03:01	N.D.	N.D.	N.D.	N.D.		
34	M12076	45	M	A01:01, 02:01	12577	19936	18279	13427	1	
35	m12077	41	F	A02:01, 02:20	729.5	23804.5	23739	14681.5		1
36	m12078	55	F	A 01:01, 02:01	4541	16769	20953	8656	1	
37	m12111	33	M	A 02:01, 29:02	6775.5	20351	20897	13114	1	
38	m12342	31	F	A 02:01, 68:01	11850	14771	17361	10207	1	

39	**m12829****	**45**	**M**	**A 01:01, 02:01**	**494**	**301**	**141**	**2493**		
40	m12830	35	F	A 02:01, 34:02	1622	20563	18881	13020	1	
41	m12859	64	M	A 02:01, 68:01	13854	6510	6567	8736	1	
42	m12876	31	F	A 02:01	9223	19572	18016	10939	1	
43	m13331	35	F	A 02:01, 34:02	9223	19572	18016	10939.22222	1	
44	m13587	54	F	A01:01, 02:01	10774	24704	17215	14824.5	1	
45	M13620	34	F	A 02:01, 02:05	8858	16278	17475	6046	1	
46	M13737	24	F	A02:01, 30:01	12351	10640	13742	13851	1	
47	M13745	73	F	A 02:01	334	27059	21690	18581		1
48	M13817	57	M	A02:01, 33:01	5137	22373	25787	8844	1	
49	M13841	40	F	A 02:01, 23:CJT	8317	26376	20218	17780	1	
50	M13874	61	F	A 01:01, 02:01	4877	28778	27177	15158	1	
51	M13994	38	M	A 01:01, 02:01	13385	21878	20418	9569	1	
Summary	median	49.0	F: 34						35	13
	Q1	37	M:17							
	Q3	60								

**** Removed fromt the cohort**

Other blood donors

1	M09011	unknown	unknown	A 02:01, 30:01	4077	19375.75	19658.5	6904	1	
2	M09478	unknown	unknown	A 02:01, 68:02	10463.5	14295	12852	7102.5	1	
3	M10315	unknown	unknown	A 02:01, 74:01	12577	19936	18279	13427	1	
4	M10475	unknown	unknown	A 02:01, 03:01	8385	14599	13762	7459	1	
5	M10942	unknown	unknown	A 02:01, 30:02	12619	19101	16101.5	13837.5	1	
6	M10999	unknown	unknown	A02:01, 36:01	8522.5	16161	16378	11837	1	
7	M11115	unknown	unknown	A 02:01, 11:01	1909.5	11804	13782.5	9916	1	
8	M11116	unknown	unknown	A 02:01, 32:01	13302.5	17015.5	16349	6881	1	
9	M11648	unknown	unknown	A02:01, 26:01	4541	16769	20953	8656	1	
10	M11774	unknown	unknown	A:02:01, 23:CJT	N.D.	N.D.	N.D.	N.D.		
11	M11852	unknown	unknown	A 02:01, 24:02	10147.5	15635	17059	4930.5	1	
12	M12104	unknown	unknown	A 02:01, 11:1	878	18500.5	21752	6652		1
13	M12105	unknown	unknown	A 02:01, 68:02	6082	18619	17102.5	9629	1	
14	M12610	unknown	unknown	A 02:1, 34:02	9173	11142	11302	8692	1	
15	M00248	unknown	unknown	A 01:02, 02:01	14400	18021	15706	13050	1	
									15	1
